# Elucidation of Melanogenesis Cascade for Identifying Pathophysiology and Therapeutic Approach of Pigmentary Disorders and Melanoma

**DOI:** 10.3390/ijms21176129

**Published:** 2020-08-25

**Authors:** Tokimasa Hida, Takafumi Kamiya, Akinori Kawakami, Jiro Ogino, Hitoshi Sohma, Hisashi Uhara, Kowichi Jimbow

**Affiliations:** 1 Department of Dermatology, Sapporo Medical University School of Medicine, Sapporo 060-8543, Hokkaido, Japan; hidat@sapmed.ac.jp (T.H.); kamitaka@sapmed.ac.jp (T.K.); uharah@sapmed.ac.jp (H.U.); 2 Cutaneous Biology Research Center, Department of Dermatology, Massachusetts General Hospital, Harvard Medical School, Charlestown, MA 02129, USA; akawakami1@mgh.harvard.edu; 3 Department of Pathology, JR Sapporo Hospital, Sapporo 060-0033, Hokkaido, Japan; jiroogino@gmail.com; 4 Department of Biomedical Engineering, Sapporo Medical University School of Medicine, Sapporo 060-8556, Hokkaido, Japan; sohma@sapmed.ac.jp; 5 Institute of Dermatology & Cutaneous Sciences, Sapporo 060-0042, Hokkaido, Japan

**Keywords:** melanogenesis, tyrosinase, tyrosinase related protein (TYRP), vesicular transport, melanosome, eumelanin, pheomelanin, pigment-type switching, hypomelanosis, melanoma

## Abstract

Melanogenesis is the biological and biochemical process of melanin and melanosome biosynthesis. Melanin is formed by enzymic reactions of tyrosinase family proteins that convert tyrosine to form brown-black eumelanin and yellow-red pheomelanin within melanosomal compartments in melanocytes, following the cascades of events interacting with a series of autocrine and paracrine signals. Fully melanized melanosomes are delivered to keratinocytes of the skin and hair. The symbiotic relation of a melanocyte and an associated pool of keratinocytes is called epidermal melanin unit (EMU). Microphthalmia-associated transcription factor (MITF) plays a vital role in melanocyte development and differentiation. MITF regulates expression of numerous pigmentation genes for promoting melanocyte differentiation, as well as fundamental genes for maintaining cell homeostasis. Diseases involving alterations of EMU show various forms of pigmentation phenotypes. This review introduces four major topics of melanogenesis cascade that include (1) melanocyte development and differentiation, (2) melanogenesis and intracellular trafficking for melanosome biosynthesis, (3) melanin pigmentation and pigment-type switching, and (4) development of a novel therapeutic approach for malignant melanoma by elucidation of melanogenesis cascade.

## 1. Introduction

Melanin determines colors of our skin, eyes, and hair and protects the skin from sunlight exposure. Melanin is a biopolymer synthesized within pigment cells, melanocytes, and packaged in the specialized lysosome-related organelles called melanosomes. The biological and biochemical pathway of melanin biosynthesis is known as melanogenesis. Melanogenesis is an enzymatic reaction in which melanin-forming tyrosinase family proteins convert an amino acid, tyrosine, to form brown-black eumelanin and yellow-red pheomelanin in melanocytes [1]. Melanogenesis involves a series of paracrine and autocrine signals, beginning with activation of the cyclic adenosine monophosphate (cAMP)/cAMP response element-binding protein (CREB)-signaling pathway in melanocytes, mainly by a G-protein-coupled receptor, melanocortin 1 receptor (MC1R) in response to its ligands, mainly alpha-melanocyte-stimulating hormone (α-MSH). Activated MC1R increases intracellular cAMP, which in turn activates CREB and eventually stimulates transcription of microphthalmia-associated transcription factor (MITF), a master transcription factor of melanocyte development and differentiation. MITF activates expression of key melanin-forming genes including genes for tyrosinase family enzymes and the melanosomal structural matrix protein PMEL. MC1R loss-of-function variants result in fair-colored skin and hair [2].

Tyrosinase is a glycoprotein that acts as the major player of this melanogenesis pathway. Its glycosylation process is regulated by a number of molecular chaperons, including calnexin in the endoplasmic reticulum [3,4]. Vesicular transport occurs to carry tyrosinase and its related proteins from the trans-Golgi network to melanosomal compartments. In addition, small molecular materials that may be directly or indirectly involved in melanogenesis accumulate on the cell surface of melanocytes and are endocytosed through vesicular transport to melanosomal compartments [5]. A large number of transporters, such as small GTP-binding proteins, adaptor proteins, and phosphoinositide-3-kinase (PI3K) are involved in vesicular transport for melanosome maturation [6,7,8,9,10]. Thus, the sorting motifs of tyrosinase family enzymes and other melanosomal proteins are unique and complex. Once biosynthesis of eumelanin or pheomelanin is completed, eumelanogenic melanosomes become ellipsoidal granules with specific lattice-like internal structures, while pheomelanogenic melanosomes take oval forms with many granular internal substructures. The mature melanosomes move along dendritic processes and are transferred to surrounding keratinocytes [1]. Fully melanized melanosomes are finally delivered to keratinocytes throughout the skin. The symbiotic relation between a melanocyte and an associated pool of keratinocytes is called an epidermal melanin unit (EMU). Diseases involving alterations of EMU show pigmentation phenotypes such as oculocutaneous albinism and Hermansky–Pudlak syndrome [11].

This review discusses certain aspects of current progresses in major pathways of melanogenesis cascade which include (1) melanocyte development and differentiation, (2) melanosome biosynthesis and intracellular trafficking, and (3) melanin pigment-type switching, and then introduces the possibility of applying the knowledge of melanogenesis cascade for elucidating the pathophysiology of pigmentary disorders and developing a novel therapeutic approach for malignant neoplasia of melanocytes, melanoma [12].

## 2. Melanocyte Development and Differentiation

### 2.1. Melanocyte Development from Neural Crest

Neural crest cells are migratory multipotent cells derived from the dorsal neural tube during embryonic development that give rise to neurons, glial cells, bone and cartilage cells, and melanocytes. The “classic” melanocyte precursors, melanoblasts, migrate in the dorsolateral pathway to reach the basal layer of epidermis and hair follicles [13]. Adameyko et al. found multipotent Schwann cell precursors to be a source of melanocytes [14]. Schwann cell precursors produce the majority of body and extremity melanocytes [14], while both “classic” melanocyte precursors and Schwann cell precursors produce cranial melanocytes [15]. Apart from the skin and hair, melanocytes exist in the eye, inner ear, and internal organs such as lung, heart, and aorta. Coat color mutants in mice and other species (e.g., *Kit*, *Ednrb*, and *Edn3*) led researchers to identify genes involved in melanocyte development [16,17,18]. Melanocyte stem cells localize in the bulge region of hair follicles and produce epidermal and hair follicle melanocytes [19]. Reactive oxygen species induced by genotoxic stresses and pain-induced adrenaline cause hair graying by making melanocyte stem cells differentiate [20,21,22]. 

### 2.2. A Master Role of MITF in Melanocyte Development and Differentiation

MITF belongs to the basic helix-loop-helix leucine zipper transcription factor family and its mutations in mice cause coat color dilutions and melanocyte loss [23,24,25]. In humans, *MITF* mutations cause Waardenburg syndrome type 2A, which phenocopies melanocyte loss [26]. MITF binds a consensus DNA binding motif E-box (CA[C/T]GTG) and regulates gene transcription by making homodimers or heterodimers with other MiT family members TFE3 and TFEB [27]. KIT increases both transcriptional activity and degradation of MITF by phosphorylating MITF at serine 73 and serine 409 [28]. MITF turns on melanogenesis by activating transcription of pigment genes such as *tyrosinase* (*TYR*), *tyrosinase-related protein 1* (*TYRP1*), *dopachrome tautomerase* (*DCT*), and *PMEL* [29,30,31].

The cAMP/CREB- and Wnt-signaling pathways, PAX3, and SOX10 activate transcription of the melanocyte specific MITF-M isoform [32,33,34,35]. The cAMP/CREB pathway plays a key role in sun tanning and determining skin and hair colors. UV irradiation damages DNA and activates TP53, which induces proopiomelanocortin (POMC) expression in keratinocytes [36]. Secreted α-MSH cleaved from POMC binds MC1R on melanocytes, induces MITF, and activates melanogenesis. MC1R loss-of-function variants result in fair-colored skin and hair.

**Figure 1:**
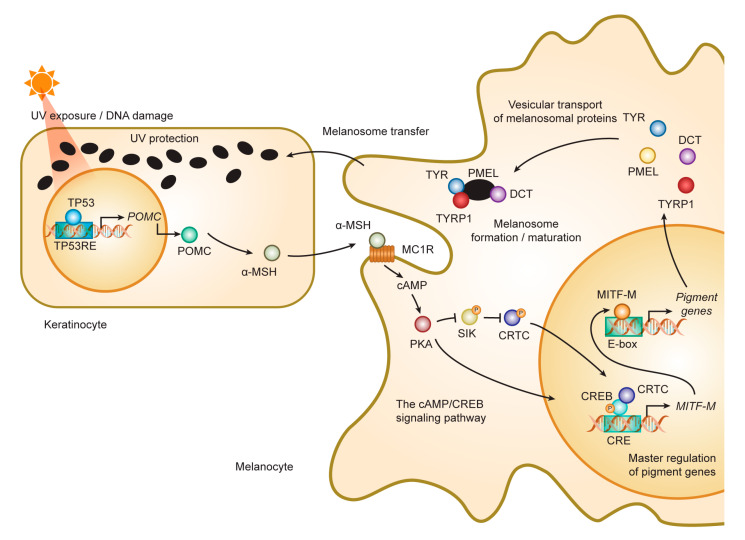
Role of MITF in melanogenesis cascade in human skin. The cascade of melanogenesis consists of the interaction between a melanocyte and an associated pool of keratinocytes, called epidermal melanin unit. MITF plays a master role in the cascade process for the activation/differentiation of the melanocyte followed by the induction of melanogenesis cascade such as during UV-induced tanning reaction. UV damages DNA in keratinocytes. DNA damage activates TP53 that initiates the transcription of *POMC*. POMC is cleaved into peptides including α-MSH. α-MSH secreted from keratinocytes binds MC1R and increases cAMP in melanocytes. PKA activated by cAMP activates CREB transcriptional activity in two ways: (i) Phosphorylate CREB; (ii) inhibiting SIK, a negative regulator of CREB co-activator CRTCs, by phosphorylating SIK. CREB activates the transcription of MITF-M. MITF-M activates the transcription of pigment genes including *TYR*, *TYRP1*, *DCT*, and *PMEL*. Melanosomal proteins are delivered to melanosomes. Matured melanosomes are transferred from melanocytes to keratinocytes and accumulate on the nucleus to block UV light. Abbreviations: α-MSH, alpha-melanocyte-stimulating hormone; cAMP, cyclic adenosine monophosphate; CRE, cAMP response element; CREB, CRE-binding protein; CRTC, CREB-regulated transcriptional coactivator; MC1R, melanocortin 1 receptor; MITF, microphthalmia-associated transcription factor; SIK, salt-inducible kinase; PKA, protein kinase A; POMC, proopiomelanocortin; TYR, tyrosinase; TYRP1, tyrosinase-related protein 1.

CREB activation rescues pigmentation from MC1R loss-of-function polymorphisms. Forskolin, which increases a cAMP level by activating adenylate cyclase, made the fair-colored skin of *Mc1r* mutant mice darker and protected skin from UV light. In addition, salt-inducible kinase (SIK) inhibitors made not only *Mc1r* mutant mouse skin but also fair-colored human skin darker by activating CREB through translocating CREB co-activator CRTC into the nucleus [37,38] (Figure 1). Although detailed mechanisms remain unclear, prohibitin 2-light chain 3-CREB axis activated melanogenesis through increasing MITF-M expression in melanocytes and melanoma cell lines [39,40]. Melanogenin and its analogues, which activate this pathway, could also rescue pigmentation. These findings suggest that we could modulate genetically defined melanogenesis activity. 

## 3. Melanogenesis and Intracellular Trafficking for Melanosome Biosynthesis

### 3.1. Formation and Maturation of Melanosomes

**Figure 2:**
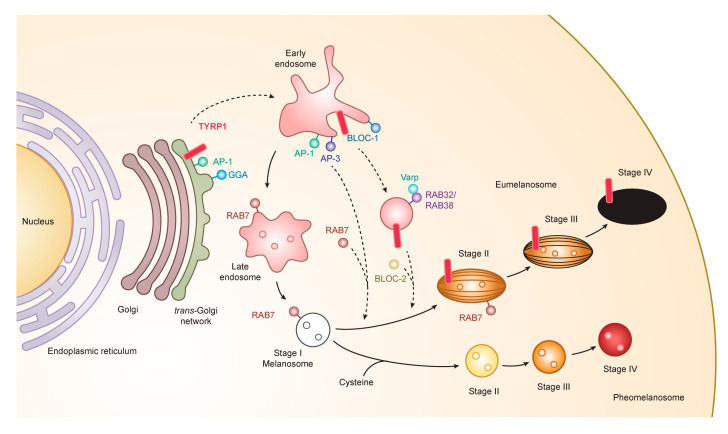
Melanogenesis and intracellular trafficking for melanosome biosynthesis. MITF turns on melanogenesis by activating transcription of pigment genes such as *TYR*, *TYRP1*, *DCT*, and *PMEL* which are transported from the trans-Golgi network and incorporated into early/late endosomal compartment to form melanosomes. Melanosomes have four maturation stages characterized by unique shapes and amounts of melanin pigments, i.e., eumelanin and pheomelanin. In the case of TYRP1, AP-1 and GGA play key roles in the sorting process of export from trans-Golgi network via early/late endosomes to stage I melanosomes by two other mechanisms: (i) BLOC-1/RAB32/RAB38/Varp/BLOC-2; (ii) AP-1 or AP-3. RAB7 is required for the stage I melanosome formation and the TYRP1 sorting from early/late endosomes to stage I melanosomes. Abbreviations: AP, adaptor protein; BLOC, biogenesis of lysosome-related organelles complex; GGA, Golgi-localized γ-ear-containing ADP-ribosylation factor -binding protein.

Melanosomes have four maturation stages characterized by unique shapes and amounts of melanin pigments. In stage I, the melanosome structure is similar to the vacuolar domain of early/late endosomes, presenting as intralumenal vesicles with the deposition of fibrils composed predominantly of the pigment cell-specific protein PMEL (also known as the silver locus product, *PMel17*), an integral membrane protein [41,42,43]. PMEL is responsible for the ellipsoidal shape of melanosomes. In stage II, the ellipsoidal form of melanosomes is morphologically changed due to reorganization of the fibrils after cleavage of PMEL and release of the Mα moiety, which is formed from the Mβ membrane fragment. Stage I and stage II melanosomes are called premelanosomes, which lack deposits of melanin; it is deposited in PMEL fibrils of stage III melanosomes. Fully melanized melanosomes are the characteristic of stage IV. During stages II to IV, melanogenesis proteins including tyrosinase, TYRP1, and TYRP2/DCT are delivered to melanosomes [44,45] (Figure 2).

In addition to PMEL, several other proteins including OA1 (GPR143), MART1, and SLC45A2 are involved in melanosome biogenesis [46,47]. OA1 (ocular albinism 1) is the G protein-coupled receptor for Dopa and tyrosine. A mutation in OA1 causes ocular albinism. Albinism refers to conditions in which there is little or no pigment synthesis due to various gene mutations. There are two categories of albinism; one is oculocutaneous albinism (OCA), in which pigment synthesis is dysfunctional in the skin, hair, and eyes. The other category is ocular albinism, in which only the eyes are affected [48,49,50] (Table 1).

### 3.2. Roles and Signaling of Tyrosinase and Related Proteins in Melanogenesis

**Figure 3:**
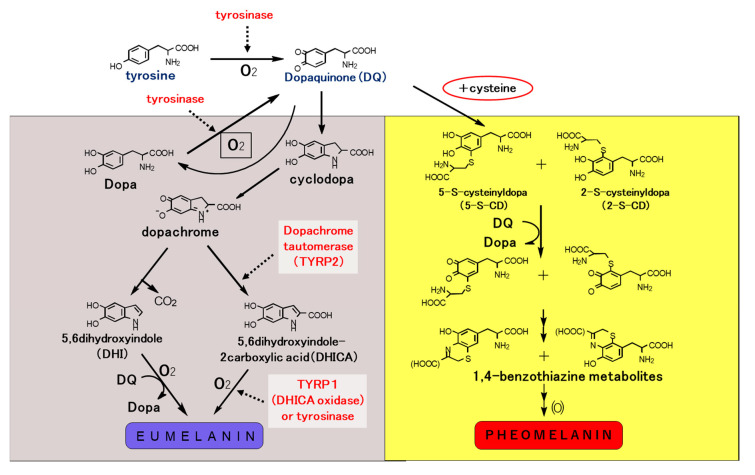
Biosynthetic pathway of eumelanin and pheomelanin. Eumelanin and pheomelanin begin with the common pathway by converting tyrosine to Dopa and subsequently Dopa to dopaquinone. Tyrosinase interacts with two tyrosinase related proteins, TYRP1 and TYRP2 or dopachrome tautomerase (DCT) to form eumelanin pigments. On the other hand, cysteine is incorporated into dopaquinone to form cysteinyldopa which is then converted to benzothiazine metabolites to form pheomelanin pigments. Dopaquinone obtained by tyrosinase oxidation is a highly reactive intermediate, and in the absence of thiol compounds, it undergoes intramolecular cyclization, leading eventually to the production of eumelanin. However, the intervention of thiols, such as cysteine, with this process gives rise to the thiol adducts. Further oxidation of these cysteinyldopa isomers leads to the production of pheomelanin. In reality, most of the melanin pigments present in the hair and skin, and in melanomas may not be homopolymers of a single monomer unit, but rather they are complex heteropolymers made up of both eumelanin and pheomelanin building blocks.

Melanocyte differentiation is characterized by melanin biosynthesis, a biological property unique to the different types of melanin-forming cells, e.g., melanophores, melanocytes, and their neoplastic counterparts, melanoma cells. Two forms of melanin pigment, i.e., brown-black eumelanin and yellow-red pheomelanin, are produced within melanosomes (Figure 3). Lower vertebrates do not synthesize pheomelanin.

**Figure 4:**
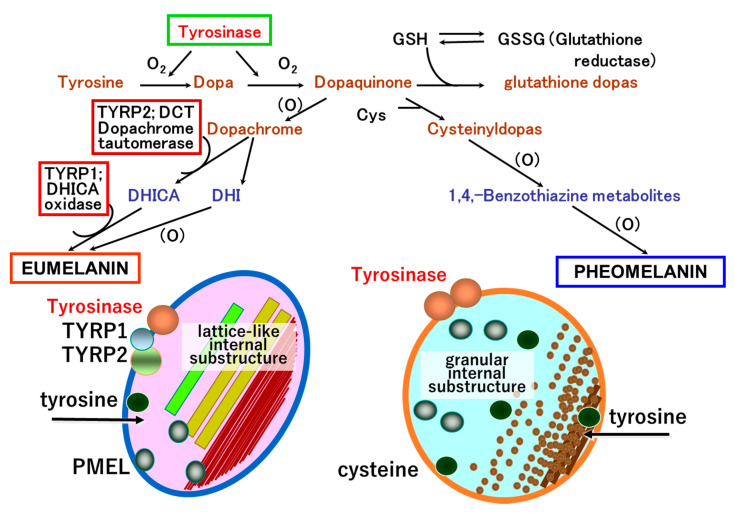
Cascade of eumelanogenesis and pheomelanogenesis. Mature stage IV melanosomes reveal a unique shape. Eumelanosomes become ellipsoidal with specific lattice-like internal structure that is formed by structural matrix protein, PMEL. Pheomelanosomes take oval forms with many internal structures. Both eumelanosomes and pheomelanosomes possess a significant number of microvesicles, which were originally reported as “vesiculoglobular bodies” deriving from the carriers of vesicular transport [41].

Tyrosinase is the key enzyme that initiates melanin biosynthesis, converting tyrosine to dopa and subsequently Dopa to dopaquinone. Tyrosinase interacts with two tyrosinase-related proteins, TYRP1 and TYRP2 (dopachrome tautomerase, DCT). These three enzymes are membrane-bound melanogenic glycoproteins expressed exclusively in melanocytes and melanoma cells. TYRP1 and TYRP2 function only in eumelanin biosynthesis, with TYRP2 catalyzing tautomerization of dopachrome to 5,6-dihydroxyindole-2-carboxylic acid (DHICA), which is converted to eumelanin by TYRP1 (Figure 4).

Tyrosinase and TYRP1 are similar in structure, having approximately 40% amino acid homology with a common NH_2_-terminal signal sequence, a cysteine-rich region, two copper-binding domains, a transmembrane region, and a cytoplasmic tail containing a dileucine motif. Tyrosinase and TYRP1 are post-translationally glycosylated and have mature protein sizes of 65–72 and 75 kDa, respectively. TYRP1 is the most abundant melanosomal protein in melanocytes. While the enzymatic activity of TYRP1 is not fully understood, murine Tyrp1 has activity as a DHICA oxidase, generating indole-5,6-quinone-carboxylic acid, a precursor of eumelanin. TYRP1 forms a stable complex with tyrosinase in the melanosomal membrane and scavenges tyrosinase-mediated oxidative species. Thus, it is likely that TYRP1 stabilizes the melanosomal membrane in melanin biosynthesis.

### 3.3. Transport to Melanosomes and Sorting of Tyrosinase and Tyrosinase-Related Proteins 

The generation of melanosomes and synthesis of melanin require multiple sorting events in the biosynthetic and endocytic pathways of melanocytes. These sorting events are highly controlled by several trafficking machineries including the interaction of heterotetrameric adaptors with specific amino acid motifs in the cytoplasmic tail of melanosomal cargo proteins. In order to synthesize melanin, melanogenic enzymes must be delivered into melanosomes. Two transport pathways are involved in transporting proteins from early/late endosomes to melanosomes. One pathway is mediated by adaptor-related protein (AP) complexes, mainly AP-3 and AP-1, and the other pathway is mediated by biogenesis of lysosomal organelle complexes 1 and 2 (BLOC-1 and BLOC-2). Many of the genes coding for AP and BLOC subunits are controlled by MITF. AP-3 subunits include both ubiquitous and tissue-specific isoforms and appear to be involved in the transport of tyrosinase from endosomes to melanosomes. Additionally, AP-1 allows the organization of tyrosinase and TYRP1 by employing the microtubule motor protein KIF13A [51,52] (Figure 2).

The other protein complexes involved in the assembly of melanogenic enzymes into melanosomes are the BLOC complexes. BLOC-1 has multiple subunits including BLOC1S1, BLOC1S2, BLOC1S3, BLOC1S5, cappuccino, pallidin, snapin, and dysbindin. Most recently, BLOC1S5, corresponding with the mouse mutation muted, was found to be the responsible gene for Hermansky–Pudlak syndrome type 11 [53]. BLOC-1 allows the delivery of ATP7A, a copper carrier protein that is essential for tyrosinase activity, to melanosomes. Deficiency of BLOC-2, which contains three subunits, leads to the impaired delivery of tyrosinase and TYRP1. BLOC-3 is another complex in this protein family, but its role in melanogenesis is not clear [54,55].

Tyrosinase and TYRP1 are transported from the trans-Golgi network (TGN) to early/late endosomal compartments of stage I melanosomes. The compartments are associated with cation-independent mannose 6-phosphate receptor (CI-M6PR) and regulated by the ADP-ribosylation factor (ARF) [56]. We have studied the mechanism for intracellular trafficking of tyrosinase and TYRP1 proteins. Our preliminary studies indicated that the interaction of tyrosinase with both AP-1 and AP-3 is likely to be involved in its transport to pre-formed immature stage II melanosomes. AP-3 has been shown to be involved in a transport step from endosomes to melanosomes, but it is unknown which trafficking step AP-1 is responsible for [57]. AP-3 does not interact with TYRP1, but AP-3 mediates tyrosinase trafficking in human melanocytes [58]. TYRP1 is transported exclusively to melanosomes, but not to lysosomes [43]. TYRP1 is shown to be co-localized with AP-1 in the vicinity of the TGN [43,58], suggesting the involvement of AP-1 in proper targeting of TYRP1 to melanosomes. AP-1 interacts with the cytoplasmic tail of TYRP1 in a yeast three-hybrid assay [57], suggesting that AP-1 interacts with TYRP1 in melanocytes.

A novel family of monomeric clathrin adaptor proteins, Golgi-localized γ-ear-containing ARF-binding proteins (GGAs), has been shown to regulate clathrin-mediated anterograde transport from the TGN independently of or in concert with AP-1 [58,59]. Three isoforms of GGA proteins (GGA1-3) have been identified [58,59,60].

### 3.4. Novel Functional Motif of TYRP1 in the Early Stage of Melanogenesis

Mutations of tyrosinase cause oculocutaneous albinism type 1. AP-3 binds the di-leucine motif in the cytoplasmic domain of tyrosinase [61]. Primary melanocytes from a patient with Hermansky–Pudlak syndrome type 2, which have a mutation in an AP-3 subunit (Table 1), were compared with normal melanocytes to show that AP-3 transfers tyrosinase (but not TYRP1) to melanosomes [62]. Both AP-1 and AP-3 sort tyrosinase from endosomes to melanosomes [57]. In our studies, it was found that the asn-gln-pro-leu-leu-thr sequence of the TYRP1 cytoplasmic domain is required for proper transportation of TYRP1 to melanosomes [63]. PI3K is necessary for TYRP1 transportation [56]. Rab7 plays a key role in transport of tyrosinase and TYRP1 from endosomes to melanosomes [6,8,64]. In addition, Rab7 is involved in the maturation of the melanosomal matrix protein PMEL [9]. It would be interesting to explore if TYRP1 may contribute in the development of a melanoma-targeted therapy or drug delivery system, in as much as melanogenesis and TYRP1 are biological properties highly elevated and uniquely expressed in melanocytes and melanoma cells.

## 4. Melanin Pigmentation and Pigment-Type Switching

### 4.1. Coat Color and Melanin Pigmentation 

The two types of melanin pigment, brown-black eumelanin and yellow-red pheomelanin, are produced exclusively by melanocytes and are mixed together regardless of the pigmentary phenotype in humans [68]. The ratio of the two melanin pigments within the melanosome is determined by race and/or specific genetic variations [69]. Black, brown, light brown, and blond hairs differ only in their eumelanin content, while red hair is exceptional in that it contains pheomelanin fourfold greater than light brown and blond hairs [70]. Variations in pigmentation derive mostly from differences in the relative amount of total melanin pigment rather than from the relative ratio of eumelanin and pheomelanin. The basal composition of the mixed eu- and pheo-melanin in an individual does not change dramatically, though external stimuli to melanocytes such as UV irradiation can affect the ratio and content of eumelanin and pheomelanin [70].

**Figure 5:**
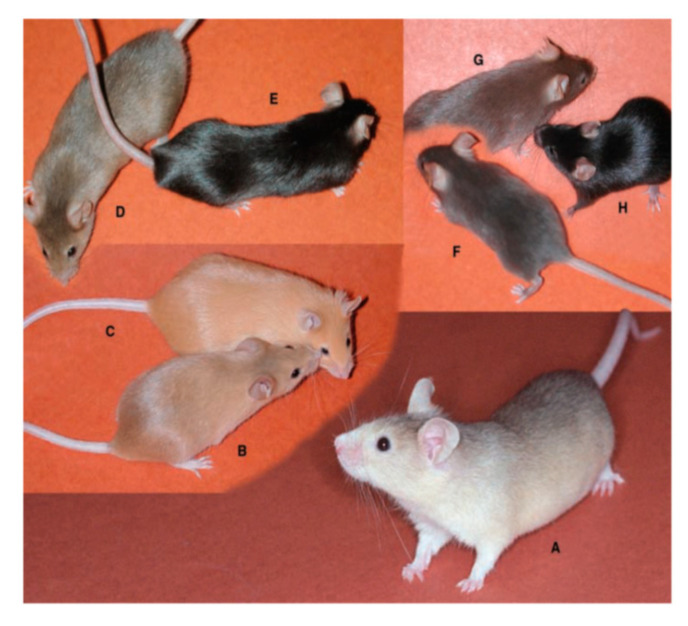
Mouse color mutations. All mice are of strain C57BL/6J except (**A**). On the C57BL/6J background, the *A^y^/a* mouse is clear yellow (**C**), whereas on the JU/CtLm background it is paler clear yellow (**A**). (**B**) *Mc1r^e^/Mc1r^e^* (recessive yellow). (**D**) *A^y^/a*, *Atrn^mg^/Atrn^mg^* (mahogany). (**E**) *a/a*, *Atrn^mg^/Atrn^mg^*. (**F**) *Dct^slt^/Dct^slt^*, slaty. (**G**) *Tyrp1^b^/Tyrp1^b^*, brown. (**H**) C57BL/6J (*a*/*a*, nonagouti), control mouse. Reproduced from reference [72], courtesy of Bennett, D.C. and Lamoreux, M.L.

On the other hand, many mammals have various patterns of hair coat color that can be changed temporally. Expression of the wild type *Agouti* gene in mice results in hair with a characteristic color pattern: subapical yellow bands in an otherwise black coat [71]. This black-yellow-black pattern results from a rapid change of melanin synthesis in hair bulb melanocytes, called pigment-type switching. In the beginning of the hair cycle (days 0–4), all hair bulb melanocytes synthesize eumelanin. During days 4–6, they produce pheomelanin in response to agouti stimuli from dermal papilla cells, followed by more eumelanin synthesis. Major regulators of pigment-type switching are the MC1R and agouti proteins. Other modulatory factors such as ATRN and MGRN1 are also involved in this process (Figure 5, Table 2). 

### 4.2. Mechanism of Pigment-Type Switching

Two major loci have a central role for pigment-type switching in mice. One is the *extension* (*E*) locus encoding the *Mc1r* gene and the other is the *Agouti* (*a*) locus encoding the *Agouti* gene (Table 2) [71]. There are dozens of mouse strains with coat color mutations in one or both of these loci. For example, sombre (*Mc1r^E-so^*/*Mc1r^+^, Mc1r^E-so^*/*Mc1r^E-so^*) and nonagouti (*a/a*) mice have black hairs in the whole body, while recessive yellow (*Mc1r^e^/Mc1r^e^*) and lethal yellow (*A^y^/a*) have yellow hairs (Figure 5) [71]. These mutant mice have contributed to the study of the mechanism of pigment-type switching.

**Figure 6:**
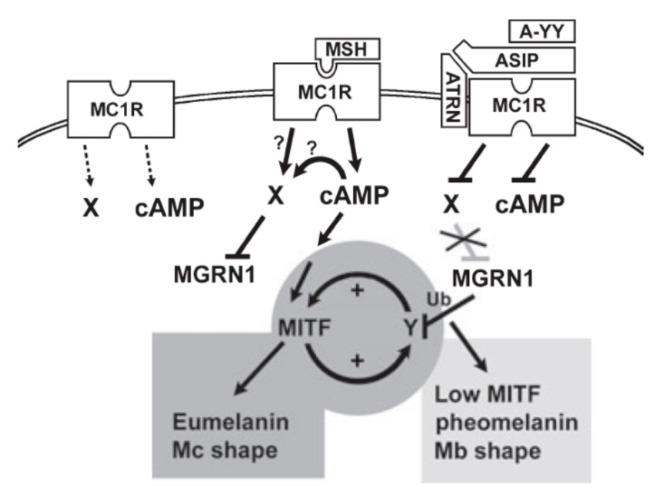
Model of pigment-type switching controlled by dual signaling from MC1R. ASIP, agouti-signaling protein (agouti protein); A-YY (ASIP-YY), a synthetic C-terminal fragment of agouti protein; Mc, melanocyte; Mb, melanoblast; Ub, ubiquitinylation. Melanocortin-1 receptor (MC1R) is shown with some basal activity (left). MC1R can activate both cAMP and another signaling pathway X. Activation of X may be cAMP-dependent or -independent. Agouti protein can antagonize cAMP signaling through its C-terminus (or as ASIP-YY) and can independently antagonize X signaling through its N-terminus and through ATRN and MGRN1, resulting in inhibition of eumelanogenesis and enhancement of pheomelanogenesis. To explain the activation of eumelanogenesis by cAMP, yet persistence of eumelanogenesis when the cAMP signal is withdrawn, a positive feedback loop was suggested. MGRN1 may break the loop by ubiquitinating and destabilizing one component Y. This would lead to a less differentiated state with low MITF activity, permissive for pheomelanogenesis. Reproduced from reference [78].

Among the 5 melanocortin receptors, only MC1R is expressed mainly in melanocytes [73,74,75]. It is a G-protein-coupled receptor, with its physiologic agonist ligands being α-MSH and adrenocorticotrophic hormone (ACTH) secreted from various cells including keratinocytes and melanocytes. UV light-irradiated keratinocytes and melanocytes produce α-MSH and ACTH from the *POMC* gene [76]. α-MSH/ACTH acts as a paracrine/autocrine mediator and binds to MC1R on the cell surface of melanocytes. MC1R also has high constitutive activity, which means that MC1R can signal without binding to an agonist [77,78]. The constitutive activity is enhanced upon paracrine/autocrine activation by α-MSH or ACTH. Activated MC1R signals through Gs proteins and adenylate cyclase and increases intracellular cAMP, which results in the activation of protein kinase A (PKA). A catalytic subunit of PKA translocates to the nucleus to phosphorylate CREB transcription factors, which increase the transcription of MITF (Figure 6). MITF increases the expression of tyrosinase, TYRP1 and TYRP2 (DCT), and the synthesis of eumelanin [75]. Moreover, MITF contributes to the control of the distribution of melanosomes and their transfer to keratinocytes by regulating the expression of RAB27A [79,80].

Agouti, another ligand of MC1R, works as a reverse agonist while α-MSH acts as an agonist. Agouti, which consists of a 109 amino acid soluble peptide and a 22 amino acid signal peptide, is secreted by dermal papilla cells in the hair follicles. The C-terminus of agouti competitively antagonizes α-MSH at MC1R and suppresses the basal activity of MC1R [81]. Inhibition of MC1R activity results in the downregulation of cAMP-PKA-CREB-MITF signaling and low tyrosinase activity. Melanocytes with low tyrosinase activity preferentially produce pheomelanin in the presence of cysteine and form a yellow band in an agouti hair [68]. Although the C-terminal domain of agouti inhibits canonical cAMP-dependent signaling, the N-terminal side of Agouti is hypothesized to be involved in cAMP-independent MC1R signaling, affecting melanocyte growth and differentiation (Figure 6) [78].

Numerous agouti alleles and mutations exist in mice, resulting in a variety of coat colors. In lethal yellow mice (*A^y^/a*), due to an upstream gene deletion, agouti is ubiquitously expressed under control of the housekeeping *Raly* promotor, which results in yellow coat color and obesity. In nonagouti mice (*a/a*), an intragenic large insertion abolishes the expression of *Agouti*, which results in a black coat color without agouti banding. In humans, the association of the *agouti signaling protein* (*ASIP*) gene, an orthologue of mouse *Agouti*, with skin/hair pigmentation has been shown [82,83].

### 4.3. Accessory Factors of Signaling Through MC1R

*Mahogany* (*mg*) is a recessive allele in mice (Table 2, Figure 5D). The gene of the *mg* locus is *attractin* (*Atrn*) and its human orthologue is the *ATRN* gene [84]. In an agouti background (*A/-*), homozygous loss-of-function alleles (*Atrn^mg^/Atrn^mg^*) reduce or eliminate pheomelanin synthesis and result in spongy degeneration and hypomyelination in the central nervous system [85]. ATRN is expressed in melanocytes as a type I membrane protein. Homozygous *Atrn^mg^* darkens the yellow hair of *A^y^/a* mice (Figure 5D), but does not affect the yellow hair of *Mc1r^e^/Mc1r^e^* mice [71]. This indicates that ATRN functions downstream of agouti and upstream of MC1R. As ATRN binds to the N-terminal domain of agouti, it is hypothesized that ATRN stabilizes agouti-MC1R binding and is involved in cAMP-independent signaling elicited by agouti [78]. ATRN is also expressed in the brain and testis and has a critical role in normal myelination in the central nervous system (Table 2).

*Mahoganoid* (*md*) is also a recessive allele. Mahoganoid mice show a phenotype similar to mahogany but also show facial dysmorphism, curled whiskers, and reduced viability due to mispatterning of the left–right axis during development [71]. The gene of the *md* locus is *Mahogunin ring finger 1* (*Mgrn1*), the product of which acts in the cytosol as an E3 ubiquitin ligase. Known targets of ubiquitination by MGRN1 are TSG101, α-tubulin, and β-arrestins [86,87,88]. TSG101 functions in the sorting of ubiquitinated transmembrane proteins into multivesicular bodies and α-tubulin is a component of microtubules that function in cell motility, morphology, intracellular transport, mitosis, and meiosis. However, the roles of TSG101 and α-tubulin in pigment synthesis are not known. Recently, it was reported that MGRN1, in the presence of wild-type MC1R, ubiquitinated β-arrestins, core components of MC1R desensitization [79]. Melanocortin receptors may also be targets of ubiquitination by MGRN1, but inhibition by MGRN1 of the functional coupling of MC1R with the cAMP pathway was independent of MC1R ubiquitination [79]. Besides its ubiquitin ligase activity, MGRN1 has the ability to bind to the cytosolic domain of MC1R competitively with G〈s and thereby inhibit the MC1R-cAMP signaling [89]. Interestingly, two of four isoforms of MGRN1 have a nuclear localization signal and may provide a pathway for MC1R signaling from the cell surface to the nucleus [89]. 

In dogs, the *K* locus dominantly affects hair color independently of the *a* locus. *K^B^* and *k^y^* are a dominant black hair and a wild-type yellow hair alleles, respectively [90]. The responsible gene of the *K* locus is *CBD103*, of which the human orthologues are the *β-defensin-3* genes *DEFB103A* and *DEFB103B*, known to encode antimicrobial proteins. The wild-type *CBD103* gene, which is responsible for the *k^y^* phenotype, encodes a secreted protein consisting of a 45 amino acid final polypeptide and a 22 amino acid signal peptide. The *K^B^* phenotype is caused by an amino acid deletion in *CBD103* (*CBD103*ΔG23). Both CBD103 and CBD103ΔG23 have affinity to dog MC1R, with CBD103ΔG23 having higher affinity. Therefore, CBD103 and CBD103ΔG23 are hypothesized to act as competitive antagonists to agouti protein [91]. In humans, β-defensin-3 inhibited α-MSH-induced cAMP production, leading to the conclusion that β-defensin-3 is a neutral antagonist [92,93]. Although it is suggested that β-defensin-3 is involved in the desensitization of MC1R, the exact role of β-defensin-3 in regulating human pigmentation needs to be elucidated.

## 5. Development of a Novel Therapeutic Approach for Pigmentary Disorders and Malignant Melanoma by Elucidation of the Melanogenesis Cascade 

### 5.1. Melanogenesis Elucidation and Therapeutic Approach for Pigmentary Disorders and Malignant Melanoma 

Malignant melanoma of skin is often associated with a fatal problem because it occurs at the highly vascular epidermal–dermal interface, and spreads from the beginning of the disease to any parts of the body more rapidly than any other solid tumor. Currently remarkable progresses have been made in developing various treatment options for metastatic melanoma. The options include checkpoint inhibition immunotherapies, e.g., anticytotoxic T lymphocyte antigen 4 (CTLA-4), anti-programmed death 1 (PD-1), molecular-targeted therapies, e.g., BRAF and MEK inhibitors, and gene directed enzyme prodrug therapy using, for example, engineered mesenchymal and neural stem cells. These treatments have shown not only high response rates, but also side effects and limitations [94,95].

Elucidation of biological properties unique to melanocytes may, however, provide an additional novel approach to overcome the difficult challenge of treating pigmentary disorders and advanced-stage melanoma. This approach is based on chemical modification of tyrosinase substrates, in particular sulfur-amine analogue of tyrosine, N-acetyl-4-S-cysteaminylphenol (NAcCAP) and N-propionyl-4-S-cysteaminylphenol (NPrCAP). NAcCAP and NPrCAP were selected because they were most efficient tyrosinase substrates that we have tested [96]. 

**Figure 7:**
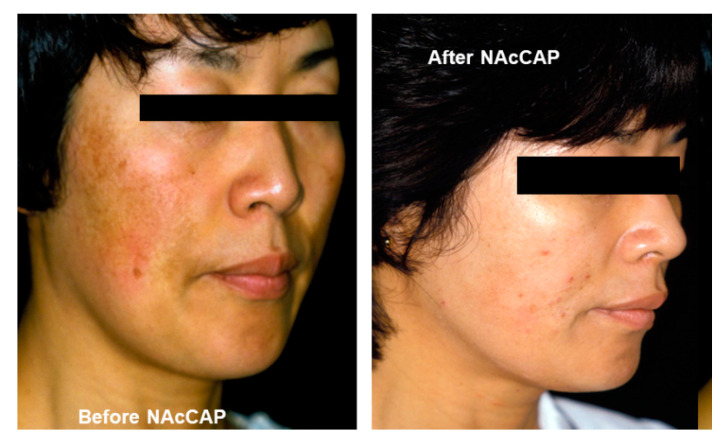
Depigmenting effect of *N*-acetyl-4-*S*-cysteaminylphenol (NAcCAP). Marked depigmentation of facial pigmentation in a melasma patient after daily topical application of NAcCAP ointment [96].

In addition, our previous studies showed that NAcCAP was selectively delivered to B16 melanoma cells in an in vivo syngeneic mouse model. We also showed a decrease of lung metastases after treatment with NAcCAP [97]. NPrCAP was also found to elicit apoptotic cell death selectively and efficiently in melanocytes and melanoma cells through production of highly reactive free radicals by reacting with tyrosinase [98]. Accordingly, our past studies have indicated that derivatives of tyrosinase substrates can provide a novel drug delivery system and selective cytocidal activity to melanocytes and melanoma cells, hence providing the basis for the development of a new therapeutic approach for pigmentary disorders such as for facial hyperpigmentation (melasma) [96] (Figure 7). However, these synthetic compounds alone may not be sufficient to control the growth of melanoma cells, indicating the necessity for an additional strategy to enhance our melanogenesis-targeting melanoma therapy. 

### 5.2. Melanogenesis-Targeted Melanoma Treatment Based on Chemotherapy and Thermo-Immunotherapy

Recently, thermal-induced anti-tumor immunotherapy utilizing magnetic nanoparticles has been applied to a number of malignant tumors [99]. Hyperthermia produces not only heat-mediated cell death but also an immune reaction due to the generation of heat shock proteins (HSPs). HSP expression induced by hyperthermia has been shown to be involved in tumor immunity. However, magnetic nanoparticles alone do not have homing ability to the targeted lesions; hence, a more direct approach is required. Conjugation of targeting materials, such as antibodies or aptamers, to the surface of the nanoparticles has been attempted to improve the delivery system. Considering the above, it was thought that NPrCAP could be a targeting candidate suitable for conjugation with the nanoparticles, due to its unique properties such as small molecular weight (MW = 225.31), targeting effect and cytotoxic ability specific to melanoma cells. Based on these findings, extensive efforts have been made to develop a novel melanoma-targeted chemo-thermo-immunotherapy (CTI therapy).

**Figure 8:**
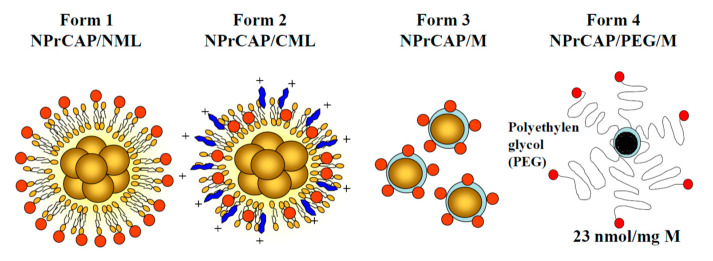
Synthesis of *N*-propionyl-4-*S*-cysteaminylphenol/magnetite nanoparticles. Several forms of *N*-propionyl-4-*S*-cysteaminylphenol (NPrCAP)/magnetite (M) nanoparticles conjugates have been synthesized. Form 1: NPrCAP is conjugated on the surface of neutral magnetic liposome particles (NML). Form 2: NPrCAP is embedded in cationic magnetic liposome particles (CML). Form 3: NPrCAP is directly conjugated with magnetite. Form 4: NPrCAP is conjugated with magnetite by polyethylene glycol (PEG).

As the initial step, several forms of NPrCAP conjugated with magnetite nanoparticles were synthesized. Figure 8 shows the conjugation of magnetites and NPrCAP. 

**Figure 9:**
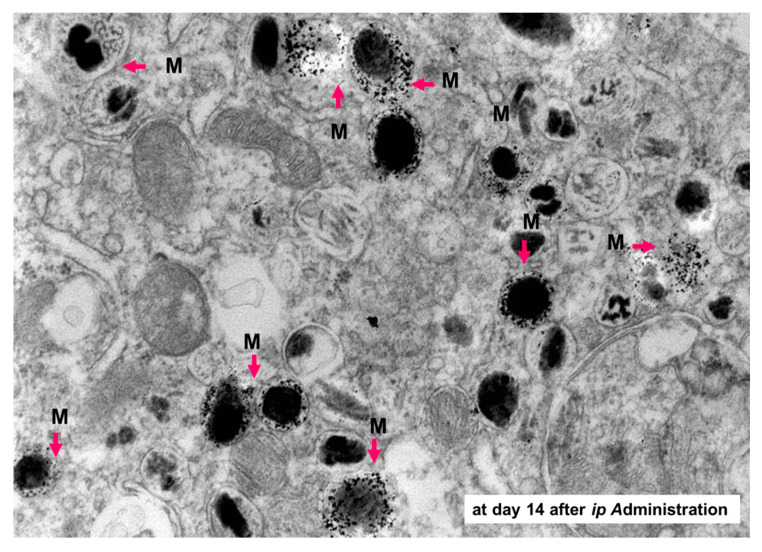
Aggregation of NPrCAP/M in melanosomes of B16 melanoma cells. The electron microscopic examination shows selective delivery of NPrCAP/M into melanosomes 14 days after intraperitoneal administration. M, magnetite.

It was found that the direct conjugate of NPrCAP with magnetite nanoparticles (NPrCAP/M) is preferentially incorporated into melanoma cell lines compared with magnetite nanoparticles alone [100]. This preferential uptake was not observed in non-melanoma tumor cell lines. We also demonstrated selective delivery and accumulation of NPrCAP/M in the in vivo mouse model. Two cell lines, B16 melanoma cells and RMA lymphoma cells, were inoculated subcutaneously and separately into the lateral flanks. Fourteen days after intraperitoneal (*i.p.*) administration, NPrCAP/M was found to have been delivered into melanoma cells, but not into lymphoma cells. Electron microscopic examination revealed NPrCAP/M nanoparticles aggregated in melanosomal compartments in the melanoma cells (Figure 9). NPrCAP/M appeared to be selectively aggregated on the cell surface of melanoma cells through a still unknown surface receptor and then incorporated into early and late endosomes of melanoma cells by the vesicular transport system. The conjugates were then incorporated into the melanosomal compartment as the stage I melanosomes derived from late endosome-related organelles, to which tyrosinase was also transported from the trans-Golgi network by vesicular transport (Figure 2). Magnetite nanoparticles (NPrCAP/M) that selectively targeted melanoma cells and accumulated in melanosomal compartments through the selective drug delivery property of NPrCAP were expected to elicit a highly efficient thermal reaction (a thermotherapy effect) after exposure to alternative magnetic field (AMF) irradiation [101]. It was also expected that NPrCAP, because of its heat resistance and stability, can also work as a chemotherapy agent during this “heat shock” process.

### 5.3. Development of Therapeutic Protocol for Melanoma Chemo-Thermo-Immunotherapy 

In order to further improve the CTI strategy, thermal immunotherapy protocols employed for other types of cancers [101] were tested and modified. In our animal study, B16 melanoma cells were inoculated subcutaneously into flanks on one side of mice and the growing melanoma masses were treated with CTI or control therapies. After treatment, the tumors were excised and the mice were rechallenged with inoculation of B16 cells into the opposite side flank, in order to assess the development of acquired tumor immunity [102]. The best anti-tumor therapeutic effect was obtained in a protocol in which direct intratumoral injection of NPrCAP/M was immediately followed by hyperthermia at 43 °C for 30 min and the procedure was repeated every other day for a total of 3 treatments. In the mice treated with this protocol, the most significant growth inhibition of both the primarily inoculated melanoma cells and the re-challenge melanoma cells was observed. In the histopathological sections, tumor necrosis was prominent and a significant number of inflammatory cells including CD4^+^ and CD8^+^ T cells were infiltrated in and around both primary treated tumors and re-inoculated tumors. Interestingly, control mice treated with only NPrCAP/M or NPrCAP, without hyperthermia, also exhibited, to some extent, growth inhibition of the re-challenged melanoma cells [103]. It was speculated that NPrCAP that remained in surviving melanoma cells after hyperthermia may provide additional acquired tumor immunity by a pathway different from hyperthermia-induced tumor immunity.

### 5.4. Acquisition of Anti-Melanoma Immunity by CTI Therapy

In our further efforts to clarify the mechanism of the hyperthermia-mediated immunity acquisition after the treatment with NPrCAP/M, it was found that NPrCAP/M with AMF irradiation induced increased expression of heat shock protein 72 (HSP72) and non-apoptotic cell death of melanoma cells [104]. HSP72 was also shown to be released extracellularly. In addition, CD8^+^ T cells were found to respond to lysates of melanoma cells treated with NPrCAP/M and AMF irradiation, and this reaction was abolished after removal of HSPs by adding anti-HSP antibody. In another mouse melanoma model to analyze the immune response of T cells induced by the CTI therapy [105], an increased level of CD8^+^ T cells was observed in swollen regional tumor-infiltrating lymph nodes (TIL) at day 14. The T cell receptor repertoire was restricted in TIL and Vβ11^+^ T cells were predominant. These findings suggested that NPrCAP/M with AMF irradiation induced an immune response through HSP, and that specific T cells responded to the treatment. The tumor infiltrating CD8^+^ T cells were found to recognize a TYRP2-derived peptide.

### 5.5. Preliminary study of CTI therapy in Advanced Melanoma Patients

**Figure 10:**
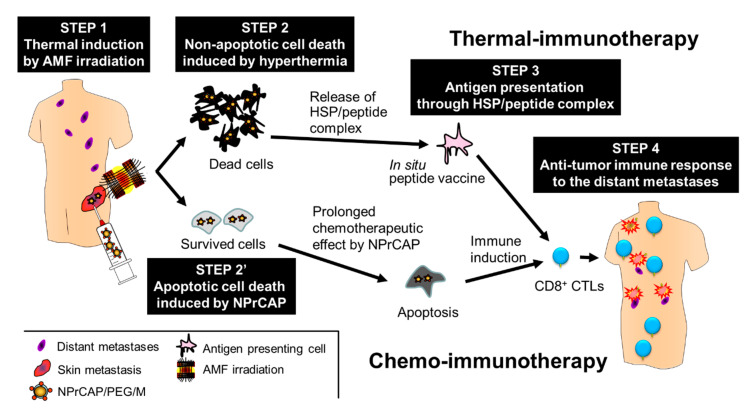
Protocol and mechanism of CTI therapy for melanoma patients in the advanced stage. STEP 1: NPrCAP/PEG/M is injected directly into the target lesion (a skin metastasis on the body surface), followed by AMF irradiation to induce intratumoral hyperthermia at 43 °C for 30 min. STEP 2: Non-apoptotic cell death is induced in the majority of melanoma cells by hyperthermia. STEP 2’: Melanoma cells surviving from the hyperthermia treatment undergo apoptotic cell death through the chemotherapeutic effect of NPrCAP. STEP 3: HSPs and antigenic peptides released from dead cells are taken up by antigen-presenting cells. STEP 4: Anti-tumor immunity acquired through hyperthermia or unknown effects of NPrCAP induces migration of CD8^+^ T cells to kill other metastases.

Based on the animal experiments, a preliminary human clinical trial of CTI therapy has been carried out for advanced melanoma patients (Figure 10). However, this preliminary clinical trial used NPrCAP/PEG/M, in which polyethylene glycol (PEG) was employed to conjugate NPrCAP with magnetite nanoparticles (Figure 8). NPrCAP/PEG/M conjugates were chemically stable, did not lose biological properties, and could be filtered and easily produced in large quantities.

A stage IV patient who had multiple skin metastases showed marked regression of other distant skin metastases and survived for 30 months. The patient died of brain metastasis. Another stage III patient also showed marked regression of regional lymph node metastases and survived for 32 months. Primarily treated skin metastases were totally excised after three rounds of the CTI therapy. The histopathological finding showed infiltration of lymphocytes and macrophages in and around the necrotic tumor tissue [12].

## 6. Ethics Approval

All clinical melanoma subjects gave their informed consent for inclusion before they participate in the clinical study. The study was conducted in accordance with the Declaration of Helsinki, and the protocol was approved by the institutional review board of Sapporo Medical University, Japan (No. 18-67).

## 7. Conclusions

As knowledge of melanin biosynthesis increases, new entities of pigmentation disorders will be identified. New therapeutic modalities for pigmentation disorders will also be developed by selectively targeting the melanogenesis cascade at different levels, thereby enabling enhancement or reduction of pigmentation in an aimed fashion with minimum side effects. The melanogenesis cascade may also be utilized to develop a novel approach for better management of hyperpigmentation disorders of the skin and malignant neoplasia of melanocytes. Our new approach of developing CTI therapy for malignant melanoma by elucidation of the melanogenesis cascade is introduced. 

## Figures and Tables

**Table 1:** Human hypomelanotic disorders associated with certain genetic defects.

Human Disease	Major Clinical Features	Mutated Gene	Gene Action in Humans
*GENERALIZED*			
Oculocutaneous albinism type 1	Hypopigmentation, nystagmus	*TYR*	Key enzyme for melanin biosynthesis
Oculocutaneous albinism type 2		*OCA2*	Melanosome biogenesis and size
Oculocutaneous albinism type 3		*TYRP1*	Melanosomal enzyme; stabilizing factor
Oculocutaneous albinism type 4		*SLC45A2*	Solute transporter; previously named *MATP*
Oculocutaneous albinism type 5		(4q24)	Responsible gene is not known
Oculocutaneous albinism type 6		*SLC24A5*	Predominant sodium-calcium exchanger in melanocytes
Oculocutaneous albinism type 7		*LRMDA*	Required for melanocyte differentiation; previously named *C10orf11*
Ocular albinism type 1	Iris hypopigmentation, nystagmus	*GPR143*	G protein-coupled receptor localized at melanosomal membrane
Hermansky–Pudlak syndrome type 1	Hypopigmentation, bleeding, immunodeficiency	*HPS1*	Component of BLOC-3, which acts as a guanine exchange factor; organelle biogenesis and size
Hermansky–Pudlak syndrome type 2		*AP3B1*	β1 subunit of AP-3 complex; organelle protein routing
Hermansky–Pudlak syndrome type 3		*HPS3*	BLOC-2 subunit 1; organelle biogenesis
Hermansky–Pudlak syndrome type 4		*HPS4*	Component of BLOC-3; organelle biogenesis and size
Hermansky–Pudlak syndrome type 5		*HPS5*	BLOC-2 subunit 2; organelle biogenesis
Hermansky–Pudlak syndrome type 6		*HPS6*	BLOC-2 subunit 3; organelle biogenesis
Hermansky–Pudlak syndrome type 7		*DTNBP1*	Dysbindin, component of BLOC-1
Hermansky–Pudlak syndrome type 8		*BLOC1S3*	BLOC-1 subunit 3
Hermansky–Pudlak syndrome type 9		*BLOC1S6*	BLOC-1 subunit 6
Hermansky–Pudlak syndrome type 10		*AP3D1*	δ1 subunit of AP-3 complex; organelle protein routing
Hermansky–Pudlak syndrome type 11		*BLOC1S5*	BLOC-1 subunit 5
Chediak–Higashi syndrome	Hypopigmentation, immunodeficiency	*LYST*	Protein required for sorting endosomal resident proteins into late multivesicular endosomes
Griscelli syndrome type 1	Hypopigmentation, pancytopenia, immunologic disorder, central nervous system abnormalities	*MYO5A*	Melanosome transport; myosin type Va/dilute mice
Griscelli syndrome type 2		*RAB27A*	Melanosome transport; RAS-associated protein/ashen mice
Griscelli syndrome type 3		*MLPH*	Melanosome transport; melanophilin/leaden mice
Phenylketonuria	Phenylalanine hydroxylase deficiency	*PAH*	Phenylalanine hydroxylase
Charcot–Marie–Tooth disease type 4J	Pale skin, alopecia, clumped melanosomes, immune effects	*FIG4*	Phosphatidyl-inositol 3,5-bisphosphate 5-phosphatase; aberrant early melanosome architecture
Menkes disease	Copper transport disorders, kinky hair	*ATP7A*	ATPase, copper-transporting α polypeptide
Wilson disease	Copper transport disorders, kinky hair	*ATP7B*	ATPase, copper-transporting β polypeptide
Cystinosis	Blond hair, multiple organ dysfunctions	*CTNS*	Cystinosin, cysteine/H^+^ symporter, which exports cysteine out of lysosomes
Tietz albinism-deafness syndrome	Congenital profound deafness, generalized hypopigmentation	*MITF*	Transcription factor; master regulator of melanocyte lineage
*CIRCUMSCRIBED*			
Waardenburg syndrome type 1 and 3	White forelock, premature graying, hearing loss, heterochromia, other neural crest defects	*PAX3*	Transcription factor; neural tube development
Waardenburg syndrome type 2		*MITF, SNAI2, SOX10*	Transcription factors; master regulator of melanocyte lineage transcription factor
Waardenburg syndrome type 4		*EDNRB, EDN3, SOX10*	Endothelin receptor B; melanoblast/neuroblast growth and differentiation factor; transcription factor
Piebaldism	White spotting, megacolon, and other neural crest defects	*KIT, SNAI2*	Receptor for SCF; required for melanoblast survival and homing; melanocyte lineage transcription factor
Tuberous sclerosis	White macules, angiofibromas and Koenen tumors	*TSC1, TSC2*	Negative regulators of PI3K-AKT-MTOR pathway
Hypomelanosis of Ito	Hypopigmentation along Blaschko lines/neural disorders	Chromosomal aberration	Somatic mosaicism probably affecting keratinocytes
Incontinentia pigmenti	White striae along Blaschko’s lines (stage 4)	*IKBKG*	Nuclear factor-κB essential modulator/inhibitor of κ light polypeptide gene enhancer in B cells, kinase γ

Modified from references of [65,66,67].

**Table 2:** Comparison of human and murine loci related to pigment-type switching.

Human Gene	Mouse Gene (Locus)	Function	Relevant Clinical Condition
*ASIP*	*Agouti (a)*	Reverse agonist of MC1R	Hair/skin color polymorphism
*MC1R*	*Mc1r (E)*	G-protein coupled receptor Hormonal regulation	Hair/skin color polymorphism Susceptibility to UV-induced damage
*ATRN*	*Atrn (mg)*	Modifier of MC1R-agouti binding	Darker hair (mouse) Spongiform encephalopathy (mouse)
*MGRN1*	*Mgrn1 (md)*	E3 ubiquitin ligase Modifier of MC1R signaling	Darker hair (mouse) Spongiform encephalopathy (mouse) Facial dysmorphology (mouse) Curled whisker (mouse)

## Abbreviations

ACTH	adrenocorticotrophic hormone
AMF	alternative magnetic field
AP	adaptor-related protein
ARF	ADP-ribosylation factor
ASIP	agouti signaling protein
ATRN	attractin
cAMP	cyclic adenosine monophosphate
CML	cationic magnetic liposome particles
CI-M6PR	cation-independent mannose 6-phosphate receptor
CRE	cAMP response element
CREB	CRE-binding protein
CRTC	CREB-regulated transcriptional coactivator
CTI	chemo-thermo-immuno
DCT	dopachrome tautomerase
EMU	epidermal melanin unit
GGA	Golgi-localized γ-ear-containing ADP-ribosylation factor-binding protein
HSP	heat shock protein
ILV	intralumenal vesicles
*i.p.*	intraperitoneal
M	magnetite
MC1R	melanocortin 1 receptor
MITF	microphthalmia-associated transcription factor
MSH	melanocyte-stimulating hormone
MW	molecular weight
NAcCAP	*N*-acetyl-4-*S*-cysteaminylphenol
NPrCAP	*N*-propionyl-4-*S*-cysteaminylphenol
NML	neutral magnetic liposome particles
OCA	oculocutaneous albinism
PEG	polyethylene glycol
PI3K	phosphoinositide-3 kinase
PKA	protein kinase A
POMC	proopiomelanocortin
SIK	salt-inducible kinase
TGN	trans-Golgi network
TIL	tumor-infiltrating lymph nodes
TYRP	tyrosinase-related protein
